# Exploiting Information Diffusion Feature for Link Prediction in Sina Weibo

**DOI:** 10.1038/srep20058

**Published:** 2016-01-28

**Authors:** Dong Li, Yongchao Zhang, Zhiming Xu, Dianhui Chu, Sheng Li

**Affiliations:** 1 School of Computer Science and Technology, Harbin Institute of Technology, Harbin, Heilongjiang, China; 2 School of Computer Science and Technology, Harbin Institute of Technology (Weihai), Weihai, Shandong, China

## Abstract

The rapid development of online social networks (e.g., Twitter and Facebook) has promoted research related to social networks in which link prediction is a key problem. Although numerous attempts have been made for link prediction based on network structure, node attribute and so on, few of the current studies have considered the impact of information diffusion on link creation and prediction. This paper mainly addresses Sina Weibo, which is the largest microblog platform with Chinese characteristics, and proposes the hypothesis that information diffusion influences link creation and verifies the hypothesis based on real data analysis. We also detect an important feature from the information diffusion process, which is used to promote link prediction performance. Finally, the experimental results on Sina Weibo dataset have demonstrated the effectiveness of our methods.

With the rapid development of networking sites (e.g., Twitter Facebook, and Sina Weibo), online social networks have drawn substantial attention. In online social networks, users can not only make friends, but also be able to seek and share information. So, online social networks support social interaction and information diffusion among users.

In these online social networks, link prediction is a critical task that not only offers insights into the factors behind creation of individual social relationship but also plays an essential role in the whole network growth. Link prediction can be applied in many fields including user recommendation (e.g., Sina Weibo introduced friend suggestions function “people you might be interested in”), community detection, network growth modeling and so on.

Link prediction has attracted extensive research attention. Nowell and Kleinberg^1^ proposed an array of methods for link prediction using network topology. They modeled a social network as a homogeneous graph, in which, each node represents a user and each link denotes a social relationship. Hasan *et al.*^2^ and Brzozowski *et al.*^3^ considered link prediction as a classification problem, they separately compared the effective of different types of features. Random walk methods^4^,^5^ and probability graphical methods^6^,^7^ also have been studied respectively in link prediction task. Some other researchers attempted to explore time feature^8^, offline events^9^, place features^10^ and spatial proximity^11^ to predict links.

Although there is so much interesting research related with link prediction, all above work ignores the impact of information diffusion process on the link creation and prediction. In online social networks, when a user observes that his neighbors share or repost a piece of information, the user will be influenced to consider whether to share or repost the information, which leads to information diffusion. Information diffusion allows users to receive or observe information that is beyond the scope of their social cycles. Furthermore, this phenomenon will influence the creation of new links. For example, there are there users *u*, *v*, and *w* in Sina Weibo (a biggest microblog media in China, currently has 500 million users). At the beginning, user *u* follows user *v*, user *v* follows user *w* and user *u* does not follow user *w*. After user *v* reposts a piece of information released by user *w*, user *u* will observe the information. If user *u* finds that the observed information is valuable or if user *u* is interested in user *w* himself in real life, then user *u* may decide to follow user *w*.

In current studies, some work has noted the above problem. Zhou *et al.*^12^, Myers *et al.*^13^, Weng *et al.*^14^ and Antoniades *et al.*^15^ analyzed the relation between information diffusion and link creation, and Zhou *et al.* also proposed a visibility-based model for link prediction. Farajtabar *et al.*^16^ did not analyze the influence of information diffusion on link creation, but directly explored information diffusion process to predict links. However, all above studies are based on the USA’s Twitter or Meme. China has the largest number of internet users in this world and social media in China usually has strong Chinese characteristics. Especially, Sina Weibo is the biggest microblog media in China, current studies^17^,^18^ have presented differences between Sina Weibo and Twitter in many dimensions (e.g., access behavior, syntactic content analysis, temporal behavior). Our work is the first to explore the influence of information diffusion on links formation from a quantitative perspective and to explore information diffusion for link prediction in Sina Weibo. Specifically, the contributions of this paper are as following:

- Considering Sina Weibo, we introduce the hypothesis that information diffusion impacts link creation and use examples to explain rationality of our hypothesis.
- We detect an important feature (observation number) from information diffusion process. Statistical analysis results in Sina Weibo dataset show that the detected feature is related to following relation creation, which verifies and supports our proposed hypothesis.
- We combine diffusion feature with network topology features for link prediction, and conduct various experiments on Sina Weibo dataset. Experiments results validate the diffusion feature is indeed helpful for promoting performance of link prediction.

The rest of this paper is organized as follows: Section 2 discusses related works; Section 3 introduces Sina Weibo dataset we collected; Section 4 introduces and verifies our hypothesis. Section 5 presents experimental results that validate the effectiveness of our methodology; Finally, Section 6 concludes.

## Related Work

Link prediction is one of the core tasks in social networks research. The basic link prediction method is based on the local neighborhood structures, as surveyed by Liben-Nowell and Kleinberg^1^,^19^. Clauset *et al.*^20^ presented a general technique for inferring hierarchical structure from network data. Yin *et al.*^21^ analyzed link structures in Twitter and proposed a novel structure-based personalized link prediction model. Random walk method is a variation of PageRank. Backstrom *et al.*^4^ proposed a supervised random walk algorithm which combines information from the network structure with node and edge level attributes to estimate the strength of social links. Yin *et al.*^5^ modeled social networks as heterogeneous graphs and applied a random walk algorithm on them to calculate link proximity.

Hasan *et al.*^2^ considered link prediction as a classification problem. They compared different classes of supervised learning algorithms based on proximity features, topological features and aggregated features. Brzozowski*et al.*^3^ also compared the effective of different types of features including user preference, user behavior and network topology. Lichtenwalter *et al.*^22^ presented a classification framework which employed their PropFlow as a feature. Wang *et al.*^6^ proposed a local probabilistic model for link prediction that used Markov Random Field (MRF), an undirected graphical model. Kashima *et al.*^7^ proposed a probabilistic model of network evolution which can be used for link prediction. Erims *et al.*^23^ coupled tensor factorization framework to predict links. Dong *et al.*^24^ tried to find missing links by convex nonnegative matrix factorization with block detection.

Zhou *et al.*^12^ found that exposing the same user multiple times does not necessarily increase the probability a new link will form, and they also proposed a visibility-based model for link prediction. Myers *et al.*^13^, Weng *et al.*^14^ and Antoniades *et al.*^15^ also focused on the same problem, but got a different conclusion that that repeated exposure to contents posted by a user increases the probability of following that user. Farajtabar *et al.*^16^ proposed a model for simulating diffusion and network events form the co-evolutionary dynamics which can be used to predict links. However, all these studies are based on the USA’s Twitter or Meme, our work attempts to explore the influence of information diffusion on link creation in Sina Weibo with strong Chinese characteristics.

In recent years, the studies on link prediction also have evolved over various aspects. One of the main aspects among these is to consider the time factor, which can be named as time-aware link prediction^8^,^25^,^26^. Besides, Leskovec *et al.*^27^ employed a logistic regression model to predict positive and negative links in online social networks. Song *et al.*^28^ explored the scalability of the proposed solutions for link prediction.

## Dataset

Sina Weibo is the most popular microblog media in China, which contains more than 500 million users as of 2015. In Sina Weibo, users can follow other users. For example, user *u* follows user *v*, we say that user *u* is a follower of user *v*, and user *v* is a followee of user *u*. If user *u* and user *v* both follow each other, we consider them mutual friends. We define *followers*(*u*) as the set of user *u*’s followers, define *followee*(*u*) as the set of user *u*’s followees and define *friends*(*u*) as the set of user *u*’s mutual friends. Sina Weibo enables users to post messages of up to 140 characters. Posting messages can contain text, pictures, videos, links and hashtag. Similar to Twitter, Sina Weibo allows users to repost or forward someone else’s messages to their followers.

The dataset we need should contain social network structure data and messages data released by users in the social network. Firstly, we select 114 seed users that are related to the internet field. Then we collect users followed by seed users and the follow relations among all these users. Secondly, we collect messages published or forwarded by all these users from 11/07/2011 to 11/28/2011. About each message, we collect id of the message, id of user posting the message, flag marking whether this message is reposted, id of reposted message and id of user posting the reposted message. Finally, we collected 30270 users, 7694408 relations, 6054000 messages with related information. The social network we collected can be modeled as a directed graph, nodes and edges in the graph correspond to users and follow relations in the social network, respectively. The statistics on the graph are summarized in Table 1.

**Figure 1:**
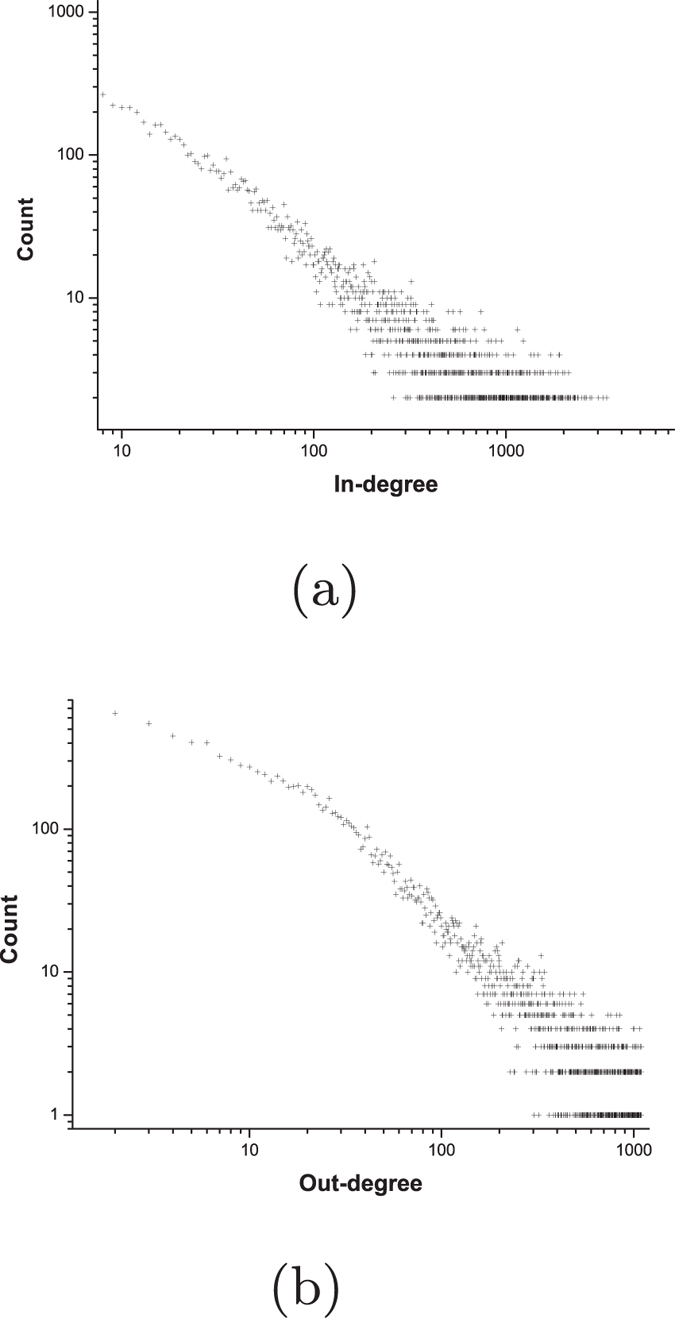
In-degree distribution and out-degree distribution of users in Sina Weibo dataset.

Figure 1 shows the in-degree distribution and out-degree distribution of users in the social network. In Fig. 1, x-axis represents in-degree or out-degree of users, y-axis represent the count of users. Each node in Fig. 1 indicates the count of users with a specific in-degree or out-degree. The in-degree distribution and out-degree distribution both exhibit heavy-tailed distributions. According to our calculations, we find that 6.0% of users receive more in-links than the other 94.0% of users and 5.4% of users receive more out-links than the other 94.6% of users. We also find that 10% of users receive about 65.7% of in-links and 10% of users receive about 69.2% of out-links.

## Information Diffusion and Link Prediction

In this section, we first introduce the hypothesis that information diffusion process impacts links creation, then use examples to explain rationality of our hypothesis. Next, we do statistics and analysis on collected Sina Weibo dataset to verify our hypothesis.

Users’ sharing or reposting behaviors create an information diffusion process that allow users to receive information from outside of their own social cycles. Our hypothesis is as follows: when one user receives or observes a piece of information released by an unrelated user, he may be interested in the information content or the user releasing the information, in that case, he may try to create a new social relation with the unrelated user. For two unrelated users, *u* and *v*, as user *u* observes more information that is released by user *v*, the probability that a new social relation from user *u* to user *v* will be created increases. We believe that the features detected from the information diffusion process will be helpful in predicting links.

**Figure 2:**
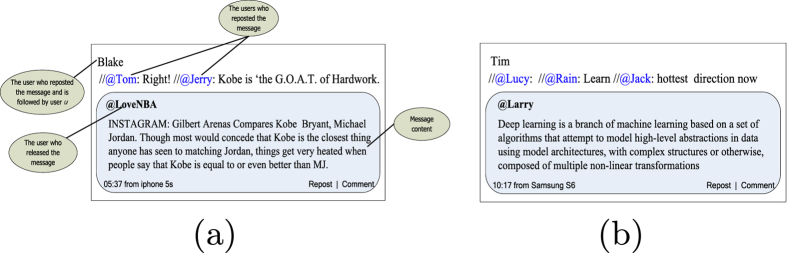
Two examples for explaining the relationship between information diffusion and follow relation.

Here, we take two examples to explain our hypothesis. Because of the copyright issues, we can not use real examples from Sina Weibo directly in this paper. Alternatively, we design and draw two examples presented in Fig. 2 following the format of Sina Weibo. The user names in examples are distributed by ourselves and do not correspond to any real user in Sina Weibo. Readers can find some similar real examples based on our examples in Sina Weibo.

We assume that there is a user *u* in Sina Weibo. Figure 2 presents two messages that user *u* observed on his homepage, which represent two situations in which user *u* may follow another unrelated user. From Fig. 2(a), we can notice that the message was released by “LoveNBA”, and Tom, Jerry and Blake reposted the message. Thus, the message was spread to user *u* according to the line of “LoveNBA → Jerry → Tom → Blake → *u*”. This message is related with basketball, and user *u* is also very interested in basketball. He liked this message, then he viewed the homepage of “LoveNBA” and found some other interesting messages. At last, he may decide to follow user “LoveNBA”. In Fig. 2(b), the message was released by “Larry” who is an expert on machine learning, and was spread to user *u* according to the line of “Larry → Jack → Rain → Lucy → Tim → *u*”. In daily life of user *u*, he is always concerned about machine learning and Mr. Larry, but he do not know Larry has opened an account in Sina Weibo. Information diffusion makes him observe the account of “Larry”, then he thinks this account is important to him and may decide to follow user “Larry”.

Information diffusion process makes one user can observe other unrelated users. For two unrelated users, *u* and *v*, we assume *D*(*u*, *v*) as the number of user *u* observing user *v*. When one of user *u*’s followees reposts one message released by user *v*, the value of observation number *D*(*u*, *v*) will increase by 1. The value of observation number *D*(*u*, *v*) is equal to the total number of behaviors of reposting user *v*’s messages performed by all followees of user *u*. Observation number is an important feature detected from information diffusion process, so here it is called as a diffusion feature. We think observation number is related to links creation and prediction. For verifying our hypothesis, we analyze the relationship between observation number and follow probability based on Sina Weibo dataset.

Firstly, we make statistics of observation number of all user pairs in Sina Weibo dataset. We get statistic results that the observation number of 87.1% of user pairs is no more than 20 times and the observation number of 97.5% of user pairs is no more than 100 times.

**Figure 3:**
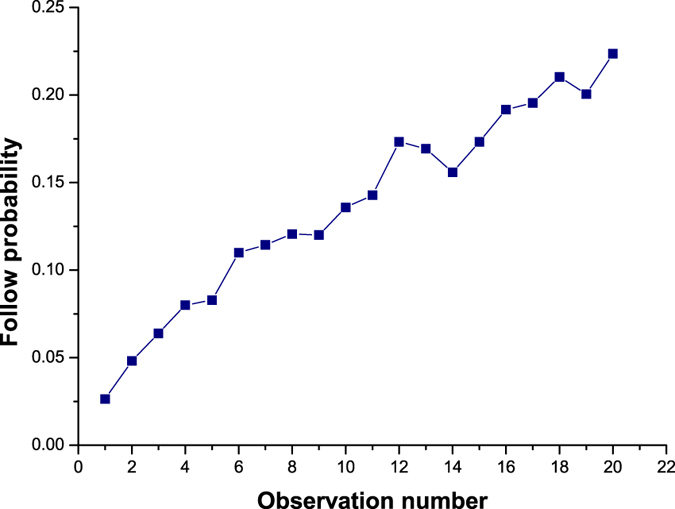
Relationship between observation number and follow probability (maximum observation number is set to be 20).

**Figure 4:**
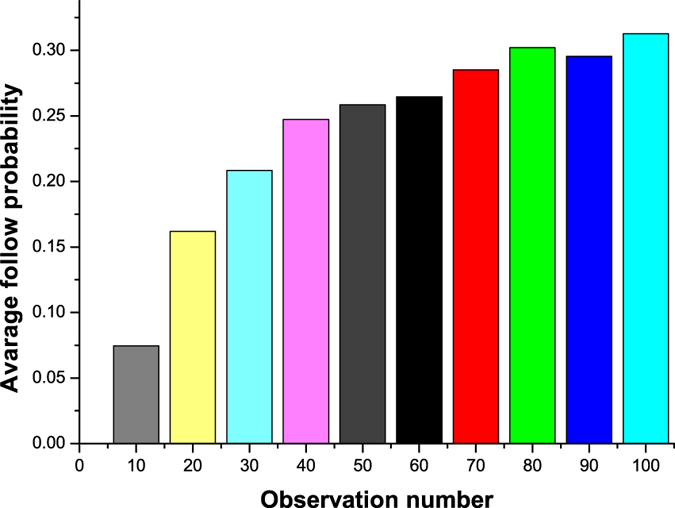
Relationship between observation number and follow probability (maximum observation number is set to be 100).

Secondly, we count the number of user pairs where a follow relation is created and the number user pairs where no follow relation is created for each observation number. We thereby calculate the follow probability for each observation number. Based on statistics about observation number in first step, we present Figs 3 and 4 whose maximum observation numbers are set as 20 and 100 respectively. In Fig. 3, the X axis represents observation number, and the Y axis represents follow probability. For representing global trend changing, we set granularity of X axis as 10 in Fig. 4. The Y axis represents the average follow probability of each 10 observation numbers. For example, the first column means that the average following probability between user pairs whose observation number is in the range of 1 to 10 is 0.014. From Figs 3 and 4, we can easily find that the probability of follow relation created is increasing with the increase of observation number. Comparing with similar studies in Twitter, our conclusion is contrary to the conclusion in^12^, however, is same with conclusions in^13^,^14^,^15^. We consider that Zhu *et al.*^12^ only examined the relationship between how often a user *v* received a specific user @CARightToKnow and the average probability that *v* followed @CARightToKnow in return. However, we test all user pairs who might form a new relationship. This may lead that Zhu *et al.* got a biased result.

Above analysis shows that information diffusion process does promote positively relation creation. Based on above explanation and analysis, we can conclude that our hypothesis is reasonable. Because information diffusion is an important driver of social relations creation, we believe that the diffusion feature (observation number) is definitely helpful in link prediction task. The diffusion feature can be combined with some other features for promoting performance of link prediction.

## Experiment

In this section, we combine the diffusion feature and topological features together for link prediction, and conduct various experiments to compare combination methods with the methods using topological features. Experiment results on Sina Weibo dataset can verify whether the diffusion feature is helpful to improve link prediction results.

### Experiment Setup

#### Prediction Setting

In our experiments, we use the Sina Weibo dataset described in section 3. We split the collected Sina Weibo dataset into training dataset and testing dataset. For each user in testing dataset, we remove half follow links, and the prediction task is then to use the pruned networks and training data to find the missing links.

#### Comparison Methods

Topological features are the most commonly used features which are detected from network topology structure^1^,^19^,^20^. In our experiment, we adopt three topological features from Sina Weibo network: mutual followers similarity, mutual followees similarity and mutual friends similarity (The definitions of followers, followees and friends can be found in section “Dataset”). Here, we take user *u* and user *v* as examples to explain three topological features. Mutual followers similarity between user *u* and user *v* is equal to 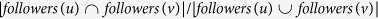. The numerator is the size of the overlap of the follower sets between *u* and *v*, and the denominator is the size of the union of the follower sets between two users which is used to normalized the numerator (mutual followers count). Mutual followees similarity and mutual friends similarity between two users are equal to 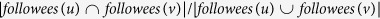, 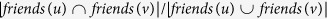 separately. The numerators and denominators of these two features are similar to these of mutual followers similarity, so we do not repeat them any more in here. Because these three topological features are decimal, we also normalize the diffusion feature for combing different features together easily.

We compare the method combing diffusion feature and topological features with other methods using single feature or combine three topological features in link prediction task. If the combination method performs better, we can conclude that diffusion feature is a helpful feature to predict links. Specifically, Following lists the methods we evaluate and compare in our experiments.

- Mutual followers similarity (method 1)
- Mutual followees similarity (method 2)
- Mutual friends similarity (method 3)
- Diffusion feature (method 4)
- Mutual followers similarity + Mutual followees similarity + Mutual friends similarity (method 5)
- Mutual followers similarity + Mutual followees similarity + Mutual friends similarity + Diffusion feature (method 6)

In our experiment, we consider link prediction task as a ranking problem. In methods 1–4, we directly assume feature values as ranking scores. Because methods 5–6 make use of multiple features, so we need combine these features together to calculate ranking scores. Here, we adopt a linear combination method. We take method 6 as an example to explain, the calculation method of the ranking score is as following:


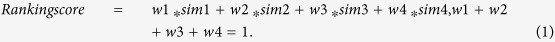


where *sim*1, *sim*2, *sim*3 and *sim*4 separately correspond to four features, and *w*1, *w*2, *w*3 and *w*4 separately correspond to the weights of four features. In this paper, we adopt the analytic hierarchy process to calculate the weight of each feature. After getting ranking scores, we select top *K* links based on ranking scores as prediction results. We evaluate the performance of different methods in terms of Precision, Recall, F1-Measure at top *K* predicted links. The values of *K* are set to be 5, 10, 15, 20, 25 and 30 separately.


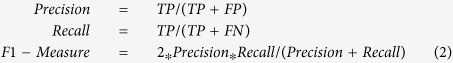


where TP stands for true positives, FN stands for false negatives, FP stands for false positive and TN stands for true negative. Under this presupposition, we denote TP as the number of links which were truly created by users and our prediction method gives the same predicting results, FP as the number of links which were not truly created by users but our prediction method predicts links will be created, TN as the number of links which were not truly created by users and our prediction method gives the same predicting results, FN as the number of links which were truly created by users but our prediction method predicts links will not be created.

### Experiment Results

**Figure 5:**
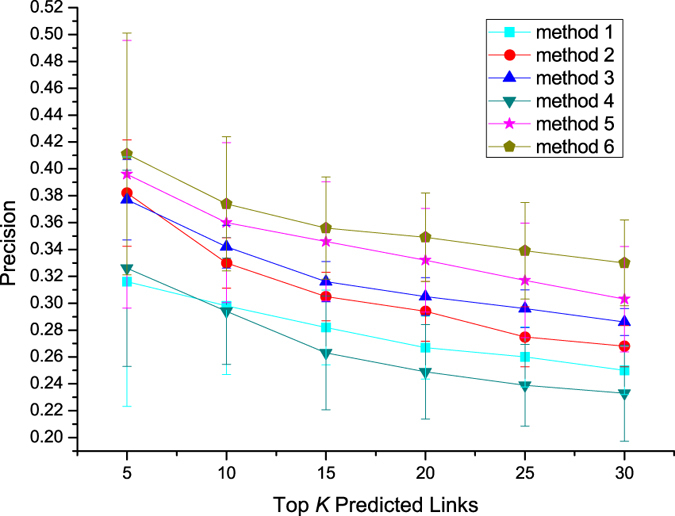
Precision of different methods on Sina Weibo dataset.

**Figure 6:**
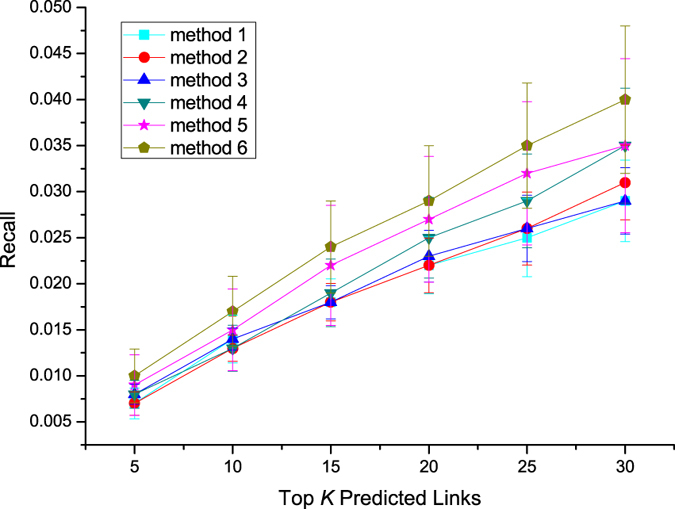
Recall of different methods on Sina Weibo dataset.

**Figure 7:**
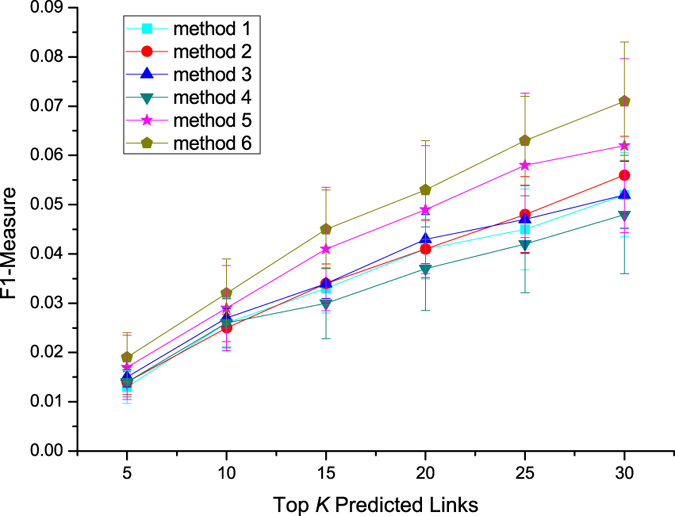
F1-measure of different methods on Sina Weibo dataset.

Figures 5, 6, 7 present the performances of these different methods by three different metrics on Sina Weibo dataset. We first analyze results of methods which make use of topological features. In the prediction task, the method 2 using mutual followees similarity performs better than the method 1 using mutual followers similarity, the method 3 using mutual friends similarity performs better than the method 2 using mutual followees similarity. Method 5, which combines three topological features, achieves better performance than do methods 1–3, each of which use a single topological feature.

The method 4 using diffusion feature can get similar performance with methods 1–3 using single topological feature. While the method 6 integrating diffusion feature and topological features together achieves best performance in our experiment. This result shows that diffusion feature is indeed a machine helpful feature in link perdition task.

To quantify the extent of fluctuations around the average, we also compute standard deviations and draw error bar for each plot in Figs 5, 6, 7. We can find that, error bars become shorter with the increase of *K* value in Fig. 5, however, become longer with the increase of *K* value in Figs 6 and 7. We also notice that, error bars of combination methods (method 5, 6) are longer than these of methods (methods 1–4) using a single feature at same *K* value. Because precision values become larger, Recall and F1-measure values become smaller, with the increase of *K* values. The evaluation metrics of combination methods are also bigger than these of methods using a single feature. So, we analyze that the changes of evaluation metric values may impact the sizes of error bars. Next, we compare two combination methods in terms of error bar and find that error bars of method 6 is shorter than these of method 5 at same *K* value. This means that the diffusion feature is helpful for getting a more smooth prediction results.

Here we should highlight that, the aid of combing diffusion feature with topological features in our experiment is to prove that diffusion feature can be used with other features together to promote prediction performance. This does not mean that diffusion feature only can be combined with topological features. In contrast, diffusion feature can be combined with any other features (e.g., user preference features, user behavior features) under any machine learning model.

## Conclusions

In recent years, online social networks have undergone a significant growth and attracted much attention. In these online social networks, link prediction is a critical task which has widespread application scenarios. In this paper, we mainly focus on Sina Weibo and point that information diffusion process affects new links creation. We detect a new feature from diffusion process and combine it with topological features for link prediction. Experimental results on Sina Weibo dataset show that new method performs better than the methods which only use topological features.

In the future, we will try to adopt classifier models to combine the diffusion feature with other features, and also will explore more helpful features for link prediction. Because the limitation of Sina Weibo APIs, our current dataset does not contain time information of link creation, so our current work is to predict whether links will be created but not to predict when link will be create. We will attempt to collect time-related datasets, and then explore the problem that how to make use of information diffusion to predict time-related links.

## Additional Information

**How to cite this article**: Li, D. *et al.* Exploiting Information Diffusion Feature for Link Prediction in Sina Weibo. *Sci. Rep.* **6**, 20058; doi: 10.1038/srep20058 (2016).

## Figures and Tables

**Table 1:** The statistics on the network graph in Sina Weibo dataset.

Nodes	Edges	Average In-degree	Average Out-degree	Maximal In-degree	Maximal Out-degree
30,270	7,694,408	248.2	219.1	6857	1096
